# Orange Dye and Silicone Glue Composite Gel-Based Optimized Impedimetric and Capacitive Surface-Type Proximity Sensors

**DOI:** 10.3390/gels9090721

**Published:** 2023-09-05

**Authors:** Khasan S. Karimov, Muhammad Tariq Saeed Chani, Noshin Fatima, Abdullah M. Asiri, Mohammed M. Rahman

**Affiliations:** 1 Faculty of Electrical Engineering, Ghulam Ishaq Khan Institute of Engineering Sciences and Technology, Topi 23640, Pakistan; 2 Center for Innovative Development of Science and Technologies of Academy of Sciences, Rudaki Ave., 33, Dushanbe 734025, Tajikistan; 3 Center of Excellence for Advanced Materials Research, King Abdulaziz University, P.O. Box 80203, Jeddah 21589, Saudi Arabia; 4 Faculty of Engineering, Technology and Built Environment, UCSI University, Kuala Lumpur 56000, Malaysia

**Keywords:** frequency response, high resolution, orange dye, silicone glue, rubbing-in technology

## Abstract

Optimized surface-type impedimetric and capacitive proximity sensors have been fabricated on paper substrates by using rubbing-in technology. The orange dye (OD) and silicone glue (SG) composite-gel films were deposited on the zig-zag gap between two aluminum electrodes fixed on a paper (dielectric) substrate. The effect of proximity of various objects (receivers) on the impedance and the capacitance of the sensors was investigated. These objects were semi-cylindrical aluminum (metallic) foil, a cylindrical plastic tube filled with water, a kopeck-shaped plastic tube filled with carbon nanotubes and a human finger. The mechanism of sensing was based on the change in impedance and/or the capacitance of the sensors with variation of proximity between the surfaces of the sensor and the object. On decreasing proximity, the impedance of the sensors increased while the capacitance decreased. The impedimetric proximity sensitivities of CNT, water, metal-based receivers and the finger were up to 60 × 10^3^ Ω/mm, 35 × 10^3^ Ω/mm, 44 × 10^3^ Ω/mm and 6.2 × 10^3^ Ω/mm, respectively, while their capacitive sensitivities were −19.0 × 10^−2^ pF/mm, −16.0 × 10^−2^ pF/mm, −16.4 × 10^−2^ pF/mm and −1.8 × 10^−2^ pF/mm. If needed for practical application, the sensors can be built in to the Wheatstone bridge, which can also increase the sensitivity of the measurement. Moreover, the sensor’s materials are low cost, while the fabrication technique is easy and ecologically friendly. The sensor can also be used for demonstrative purposes in school and college laboratories.

## 1. Introduction

Technology of proximity perception has a great potential to play an important role in the future of robotics. It has the capability to satisfy the promise of autonomous, robust and safe systems in everyday life as well as in industry, alongside humans, in remote locations underwater and in space [1]. These devices (proximity sensor) sense the presence of a physical object (also known as target) in their spatial locality. In addition to robotics, the proximity sensors have application in various industries, such as mining, manufacturing, building automation, transportation and consumer electronics. The ability of proximity sensors to gratify common application needs in safety, fault detection, access control, motion control, quality control and interactivity makes them acceptable for their extensive use [2].

Traditionally, in the sensor industry, most of the measurements are based on deformation, while, in wearable electronics, the proximity measurement (distance sensing) is becoming increasingly common. The safe interaction between environment and human being is the prime need of wearable electronics. Therefore, the detection of presence of an object without contact is very important. Until now, different types of sensors have been developed, including capacitive, infrared, ultrasonic and magnetic induction sensors. Out of various types of sensing mechanism, the proximity sensors based on capacitive sensing are more advanced than the others. The design simplicity of the device, its integration, easy readout and impartial functionality with respect to various textures and colors during detection make the proximity sensors very attractive for industrial and engineering applications.

During the last few years, different kinds of proximity sensors have been designed, fabricated and investigated. The mathematical modeling of proximity sensors and their nanocomposite-based experimental design were reported by Moheimani et al. [3]. The developed fringe capacitance model takes into account the presence of various resistivities. This model was designed for rectangular-shape sensors by using various mathematical and electrical laws. As an output, the developed model gives the distribution of electric potential that is used to calculate the capacitance (fringe) in an area of 2D domain. They developed the carbon nanotubes’ (CNTs) reinforced thermoplastic polyurethane (TPU)-based sensors with resistivity up to 10^5^. The developed sensor had a capacity to detect foreign objects in a wide range. The sensor results had a good correlation with the mathematical model. The fabrication and the investigation of a ferrous-selective sensor (proximity) for the industrial internet of things was reported by Ibrahim et al. [2]. This sensor was comprised of a force-sensing resistor and neodymium magnet. It was claimed that these ferrous-selective sensors are simple, cost effective and power efficient as compared to their counterparts.

For the detection of passive proximity, a pyramidal optical sensor (miniaturized) was described in Ref. [4]. The base of the sensor was a pyramid with light sensors on the top and the sides. This sensor was able to detect the distance and direction of the light source. The detection mechanism was based on the light intensity ratios received on the sides. The dual-mode sensors based on shared electrodes on a single polymer platform were fabricated for robotic applications [5]. These sensors were particularly important for the proximity and tactile sensing. For the robot safety control, an ultrasonic proximity sensing skin was fabricated by Tong et al. [6]. This device was based on micromachined piezoelectric (aluminum nitride) ultrasonic transducers. The design and analysis of LTCC (low temperature co-fired ceramics)-based planar inductive proximity sensors were conducted by Zuk et al. [7]. A six-layered planar coil was designed, fabricated and characterized while the electronic system was developed to process the measured values of the driving coil. These inductive sensors had the ability to measure proximity in the range of 4–7 mm.

For the detection of high range proximity, a capacitance-based sensor technology was developed by Chiurazzi et al. [8]. This system was comprised of several active units. The sensor unit (single) was made of a multilayer complaint structure with a configuration of coplanar plate capacitor. Two units (single active) were used to design the prototype. For each unit, a conductive electrode was printed on the layer of polyamide. These electrodes were positioned in the middle of insulating substrates (soft polymeric), which were also working as mechanical and electrical protection as well as shock absorbers. By the characterization of the single and multiple unit systems, it was concluded that the system with two active sensing units had 15 times more operating range as compared to single commercial capacitive sensing units of comparable dimensions.

In human-centered robotics, the proximity perception, in particular, a review on the sensing systems and applications, has also been discussed [1]. The inductive proximity sensors with differential structures have also been described [9]. The use of IoT (Internet of things)-based proximity inductive sensor for parking place availability information was presented as well in Ref. [10]. In Ref. [11], an ultra-low-power human proximity sensor using electrostatic induction was described. There are various types of proximity sensors which are in use. These sensors may include magnetic field sensors (inductive sensors), sonic sensors (ultrasonic sensors), electromagnetic sensors (infrared sensors) and electric sensors (capacitive sensors) [12].

A dual mode proximity sensor based on a capacitive and inductive sensing mode was designed, fabricated and characterized by Kan et al. [13]. These sensors can help the robot to differentiate various objects and at the same time to acquire distance information. For the plastic block detection, the capacitive sensing range was 0–5.1 mm, while, to perceive the copper block, the inductive sensing range was 0–5.6 mm. It was also concluded on the basis of a collaborative detection test that the non-ferromagnetic metals can be discriminated by the inductive mode. Similarly, the dielectric object and the ferromagnetic metals can be differentiated by the capacitive mode. Practically, these sensors could detect and separate the steel and plastic bottles. A dual mode flexible proximity sensor was designed and fabricated by Haung et al. [14]. This sensor had combined capacitive (mode-C) and resistive (mode-R) detection modes. Both modes were integrated vertically in an 8 mm × 12 mm sandwich-like chip. The silicone rubber and carbon black mixture (dielectric) was used for mode-C, while graphene nanoplatelets were used as mode-R sensing materials. The thicknesses of the mode-C and mode-R materials were 2.5 mm and 1.0 mm, respectively. The linearity of mode-R in the range of 25–65 °C is reported to be 0.998, while various objects are detected successfully in mode-C with high reversibility and fast response. To ensure the proper work of the C-mode, the effect of an object’s temperature was compensated. The combined mode study was also conducted, and it was found that, as compared to single mode sensing, the R–C dual mode sensing increased the sensing distance.

The flexible electrochemical sensors based on Al/Gr-Jelly/Cu-rubber composites were fabricated, and the effect of temperature and humidity on their voltage and impedances was studied [15]. The gel-orange dye-based flexible electrochemical cells were studied for infrared and ultraviolet irradiations sensing [16]. The orange dye-CNTs’ composite-based flexible transversal and longitudinal displacement sensors were also studied [17].

A review of these publications showed that research on flexible surface type proximity sensors based on orange dye and silicone glue composite has not been published before now. Therefore, it was considered by us; especially, the fabrication and investigation of the proximity sensors would be useful for practical applications. Research on organic composite-based surface type proximity sensors has not been conducted yet. Therefore, the fabrication of proximity sensors was undertaken to obtain some experimental data related to the development of the devices and potential applications in this area of technology, which could contribute in the future to the implementation of the results in practice. The investigations in this area of electronics could help, first of all, in the development of this area of organic semiconductor devices technology and, secondly, in their implementation in practice. Therefore, we are here presenting the results of investigations of the impedance and capacitance of the optimized surface type organic semiconductor proximity sensors. These sensors detect the proximity of objects including a semi-cylindrical metallic screen, water or CNT-filled transparent plastic tubes and the finger. The sensing mechanism of these sensors is based on the detection of changes in the impedance or capacitance between the sensing objects and the sensor.

## 2. Results and Discussion

**Figure 1:**
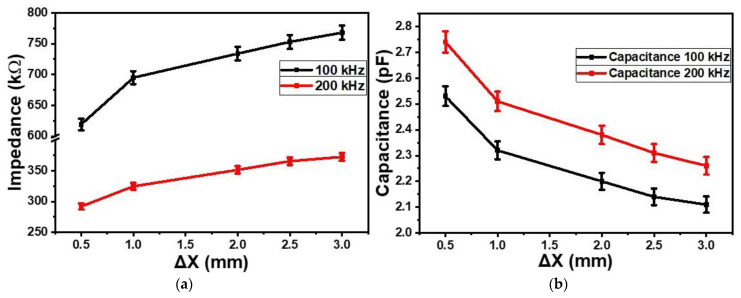
Dependences of the impedances (**a**) and the capacitance (**b**) of the sensors on the proximity (Δ*X*, mm) of CNT-based receiver at the frequencies of 100 kHz and 200 kHz.

Figure 1a shows the dependences of the impedances of the sensor on the proximity (Δ*X*, mm) or gap between the sensor and CNT-based receiver (CNT-filled kopek-shaped plastic tube) at the frequencies of 100 kHz and 200 kHz. As the proximity increased from 0.5 mm to 3 mm, the impedance of the sensor also increased up to 1.24 time at both frequencies (100 kHz and 200 kHz). Figure 1b shows the dependences of the capacitances on proximity at 100 kHz and 200 kHz. On increasing proximity from 0.5 mm to 3.0 mm, the capacitance of the sensor decreased on average by 1.20 times at 100 kHz as well as at 200 kHz. The impedimetric and capacitive sensitivities of the sensors for the CNT-based receiver went up to 59.6 kΩ/mm and 19.1 × 10^3^ pF/mm, respectively.

The proximity effect was observed between the surface of the sensor and, placed at a distance, the cylindrical plastic tube filled with water to impedances and capacitances of the sensor at 100 kHz and 200 kHz. It was found that, on increasing the gap between plastic tube separator and the surface of the proximity sensor from 0.5 mm to 3.0 mm, the impedance of the samples on average increased by 1.14 and 1.15 times at 100 kHz and 200 kHz, respectively. Under the same conditions, the capacitance decreased on average by 1.18 and 1.15 times at 100 kHz and 200 kHz, respectively. The impedimetric and capacitive sensitivities for water samples are shown in Table 1.

The dependence of the impedance of the sensor on the proximity (Δ*X*, mm) of the semi-cylindrical metallic separator (aluminum) at the frequencies of 100 kHz and 200 kHz was studied. It was found that, on increasing the gap between the metallic screen and the surface of the proximity sensor, the impedance of the sensor decreased. On changing the variable thickness of the dielectric supports (isolator) from 0.2 mm to 4.0 mm, the impedance of the samples increased on average by 1.20 and 1.26 times at 100 kHz and 200 kHz, respectively. Under the same conditions, the capacitance decreased on average by 1.19 and 1.16 times at 100 kHz and 200 kHz, respectively. The impedimetric and capacitive sensitivities for semi-cylindrical metallic samples are shown in Table 1.

**Figure 2:**
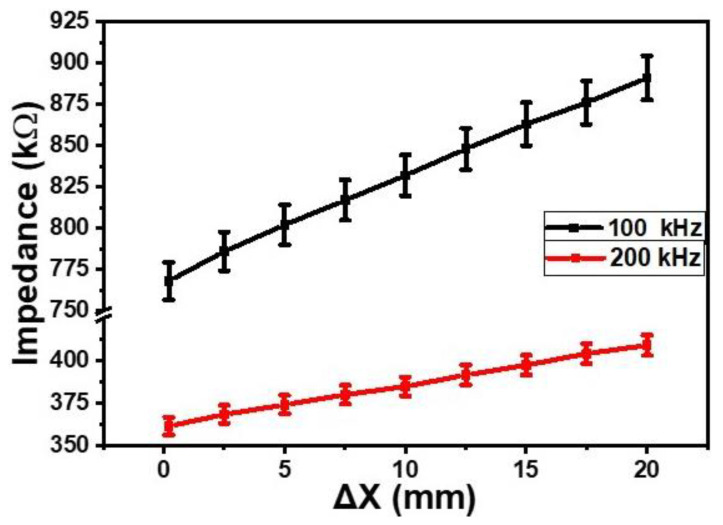
Dependences of the impedances of the orange dye and silicone glue composite gel-based sensor on the proximity (Δ*X*, mm) of finger at the frequencies of 100 kHz and 200 kHz.

**Figure 3:**
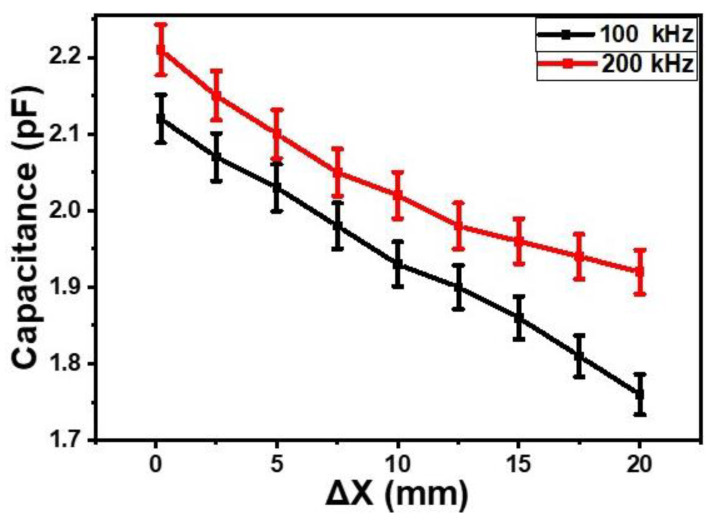
Dependences of the capacitances of the orange dye and silicone glue composite gel-based sensor on the proximity of finger at 100 kHz and 200 kHz.

The effect of finger proximity (Δ*X*, mm) on the impedance of the sensor was measured at the frequencies of 100 kHz and 200 kHz. As shown in Figure 2, on decreasing proximity (increasing distance between the finger and the sensor) from 0 to 20 mm, the impedance of the sensor increased. The average changes in the impedance at the frequencies of 100 Hz and 200 kHz were 6.2 × 10^3^ Ω/mm and 2.5 × 10^3^ Ω/mm, respectively. For the similar change in the finger’s proximity, the capacitance of the sensor also changed. Figure 3 shows the dependence of the capacitance on the proximity at 100 kHz and 200 kHz. It was found that, on increasing the gap between the finger and the surface of the proximity sensor from 0 to 20 mm, the capacitance decreased on average by 1.2 times and 1.15 times at a frequency of 100 kHz and 200 kHz, respectively. The measurements were repeated 4 to 5 times, and experimental error was found to be ±1.5% on average.

The obtained results can be qualitatively explained in the following way. By the digital meter MT 4090 LCA, the applied variable voltage to the sensor produces the variable current in the space between terminals of the meter. The value of this current and, accordingly, the impedance and the capacitance depend on the impedance between terminals of the sensor, i.e., the gap between the metallic separator and the surface of the proximity sensor or variable height dielectric supports-isolator (shown in Section 4).

Concerning the fabricated proximity sensors, the following can be mentioned: the number of advantages of the proximity sensors are well known, such as the ability to detect the movement or the presence of the objects without physical contact and relay, while, in these sensors, the information may be captured into an electrical signal. These sensors can also be defined as a proximity switch; a definition can be given to all contactless detecting sensors. The proximity sensors are nearly unaffected by the surface colors of the objects since it mainly detects physical changes. Moreover, they show suitability for the wide range of applications. Capacitive proximity sensors also have the following advantages: they are simple in construction and are adjustable; they have the ability to detect non-metallic targets and liquids; they have a low cost and high sensitivity; and they can be used for the measurement of force, pressure and humidity as well.

**Figure 4:**
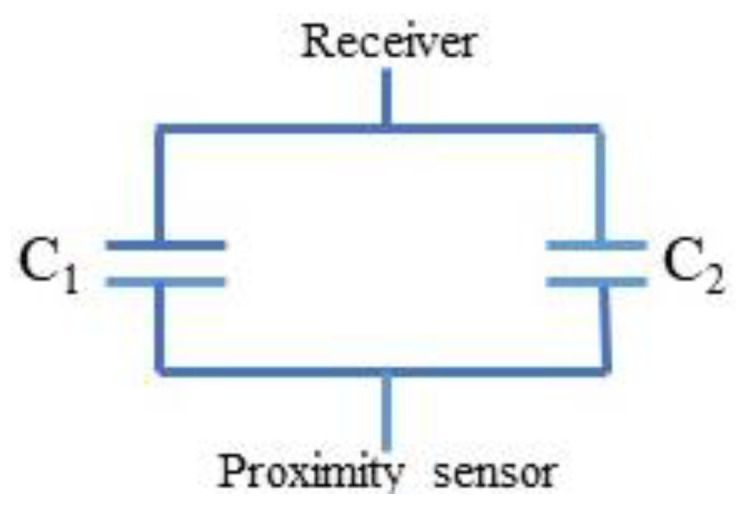
Simplified equivalent circuit of the capacitive feedback between proximity sensor and receivers.

Figure 4 shows the equivalent circuit of the proximity sensor with various receivers (CNT-filled kopek-shaped plastic tube and the plastic tube filled with water). This is the simplified equivalent circuit of the capacitive feedback between the proximity sensor and the receiver.

**Figure 5:**
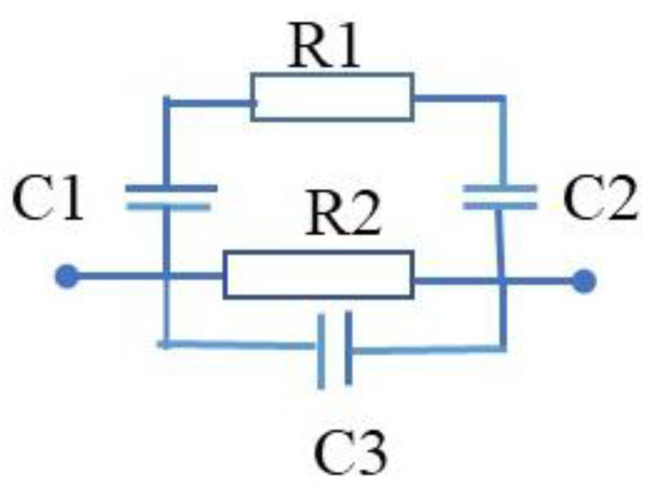
Proximity sensor simplified equivalent circuit: C1 and C2 are capacitances between semi-cylindrical metallic foil screen (R1) and proximity sensor (R2 and C3).

The equivalent circuit of the proximity sensor with the semi-cylindrical metallic receiver is shown in Figure 5. In the figure (Figure 5), the C1 and C2 are capacitances between semi-cylindrical metallic foil screen (R1) and proximity sensor (R2 and C3).

It was found that, on decreasing proximity between the sensor and the objects, the impedance of the sensor increased while the capacitance decreased and vice versa. The impedimetric sensing mechanism is based upon the changes in resistance and capacitance due to changes in mobility and charge concentration. Unlike impedimetric sensing, the capacitive sensing mainly depends upon the contribution of static charges. The impedimetric proximity sensitivities of CNT, water, metal-based receivers and the finger were up to 60 × 10^3^ Ω/mm, 35 × 10^3^ Ω/mm, 44 × 10^3^ Ω/mm and 6.2 × 10^3^ Ω/mm, respectively, while their capacitive sensitivities were −19.0 × 10^−2^ pF/mm, −16.0 × 10^−2^ pF/mm, −16.4 × 10^−2^ pF/mm and −1.8 × 10^−2^ pF/mm. Moreover, it was found that the position of the finger in the space near the proximity sensor had an influence on the impedance and the capacitance of the sensor. The results obtained about the effect of finger to the impedance and the capacitance of the sensors were comparable with the results reported in other studies [18]. The fabricated sensors showed good stability. Even after one year of fabrication, there was no significant change in the performance of these sensors.

The comparison of fabricated sensors with already reported proximity sensors in terms of materials, fabrication techniques, sensing range and sensitivity is given in Table 2.

The results obtained in this work can be simulated. For the simulation of the impedance–proximity relationship (shown in Figure 2), the following linear mathematical functions can be used [20]:

(1) $$f\left(x\right)=ax+b$$

**Figure 6:**
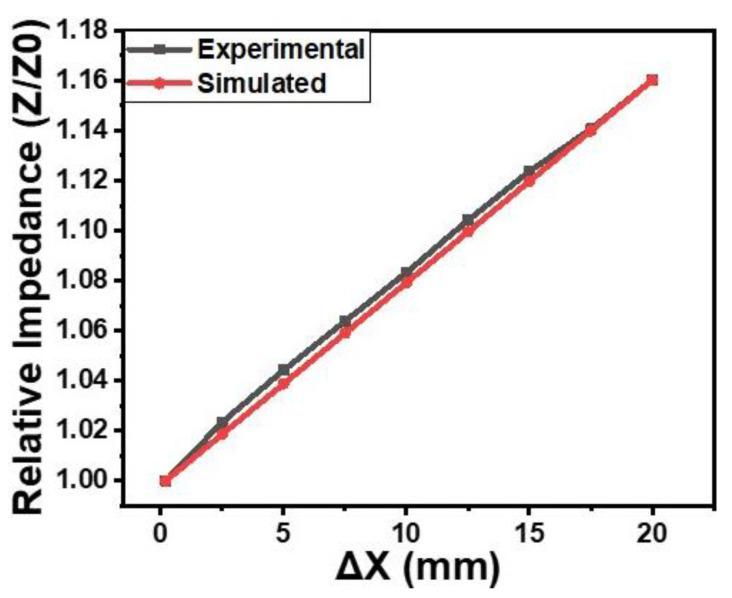
The comparison of simulated and experimental impedance–proximity (finger) relationship of the orange dye and silicone glue composite gel-based sensor.

The above mathematical function can be modified to simulate the impedance–proximity (of the finger) relationship as follows:

(2) ZZ0=∆Xk1+C

where *Z* and *Z*_0_ are the instantaneous and initial impedances, respectively. The ∆X is the proximity and *k*_1_ is the impedance coefficient of proximity. The value of *k*_1_ was calculated and found to be 0.008/mm. The *C* is the constant and its value is considered 1. The comparison of experimental and simulated results is shown in Figure 6. The simulated results are well matched with the experimental results. The mathematical relationship shown in Equation (2) can also be used to simulate the other impedance–proximity relationships.

**Figure 7:**
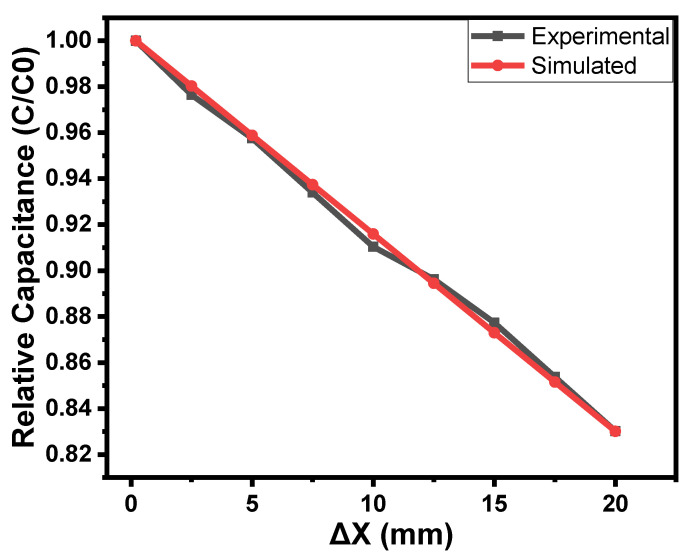
The comparison of simulated and experimental capacitance–proximity (finger) relationship of the orange dye and silicone glue composite gel-based sensor.

To simulate the capacitance–proximity (of the finger) relationship, the mathematical function shown in Equation (1) can be modified as follows:

(3) CC0=∆Xk2+d

where the *C* and *C*_0_ are the instantaneous and initial capacitance, respectively. The *k*_2_ is the capacitance coefficient of proximity and ∆X is the proximity in mm. The value of *k*_2_ was also calculated and found to be −0.0085/mm. *d* is the constant and its value is considered to be 1. Figure 7 shows the comparison of the simulated and experimental results. The simulated results are in good agreement with the experimental results. To simulate the other capacitance–proximity relationships, the mathematical function shown in Equation (3) can be used.

This paper presents the design of the optimized impedimetric and capacitive surface type organic semiconductor proximity sensor. The sensor was tested (by using a particularly designed setup), and it was found that the proximity sensor could be used for demonstrative purposes in educational institutes. The calibration of the sensors can allow the use of these devices as proximity sensors, which would be simple, low cost and reliable for utilization. However, during the last years, several papers were published in this area of technology, such as the proximity sensor (compliant ultrasound) integrated with the capability of tactile sensing for the innocuous operation of human-friendly robots [21], integrated contact and force and proximity sensing using elastomer-embedded proximity sensors [22]. For the recognition of bathroom activity, highly accurate proximity (infrared) sensors were described in Ref. [23]. RNNs-based capacitive proximity sensors for the continuous gesture (touch) recognition were presented in Ref. [24]. A novel parallel plate capacitance proximity sensor was described in Ref. [25]. An effective theory-based modelling of capacitive (parallel double-plate) proximity sensor was presented in Ref. [26]. The data published in these papers and presented by us in this paper allow us to make the following conclusion.

## 3. Conclusions

Analysis of the fabricated and investigated orange dye-silicone glue composite-gel-based flexible surface type proximity sensors allows us to consider that it is low cost, easy to manufacture and technologically and ecologically friendly. The effect of proximity between the surface of the sensor and the receivers on the impedance and capacitance of the sensor was investigated at the frequencies of 100 kHz and 200 kHz. The receivers were CNT (filled in “kopeck” shaped plastic tube), water (filled in transparent plastic tube), metal (metallic semi-cylindrical screen) and the finger. The mechanism of sensing was based on the change in impedance and/or the capacitance of the sensors with variation of proximity between surface of the sensor and the object (receiver). Moreover, it was observed that, on increasing the gap (decreasing proximity) between the receivers and the surface of the proximity sensor, the impedance of the sensor was increased, while the capacitance decreased. The sensor also can be used for demonstrative purposes in educational and industrial training institutes. The sizes of the sensors and materials which were used for the fabrication of the devices can be changed according to practical demands or their availability. That is an important factor for the utilization of the devices in practice. We believe that the information presented here will be useful for the development of the area of technology based on organic gels.

## 4. Materials and Methods

**Figure 8:**
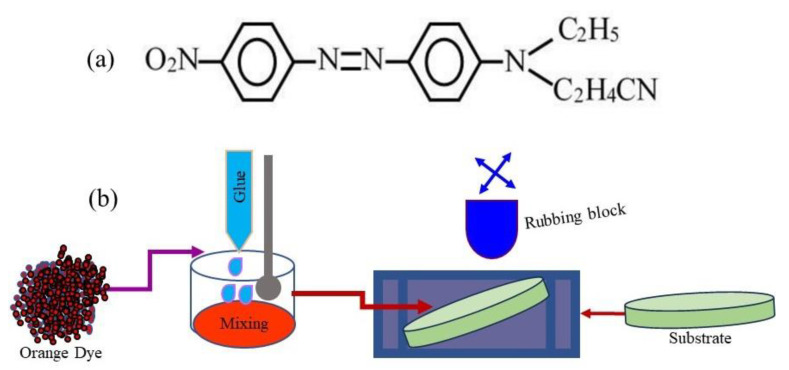
(**a**) Molecular structure of orange dye (C_17_H_17_N_5_O_2_) and (**b**) schematic diagram of the rubbing-in process.

The orange dye (OD) with 95% dye content was purchased from Sigma Aldrich (St. Louis, MO, USA, CAS Number 31482-56-1), while the silicone glue was commercially available. Figure 8a shows the orange dye’s molecular structure. As an organic semiconductor material, the orange dye (C17H17N5O2) has a density of 0.9 g/cm^3^ and its molecular weight is 323.35 g/mol, while its conduction is p-type. The IUPAC name of the orange dye is 3-[N-Ethyl-4-(4-nitrophenylazo)phenylamino]propionitrile. The silicone glue used in this work was made by UHU, GmbH & Co. KG Hermannstraße 7D—77815 Buhl/Baden Germany (www.UHU.com, accessed on 17 December 2021).

**Figure 9:**
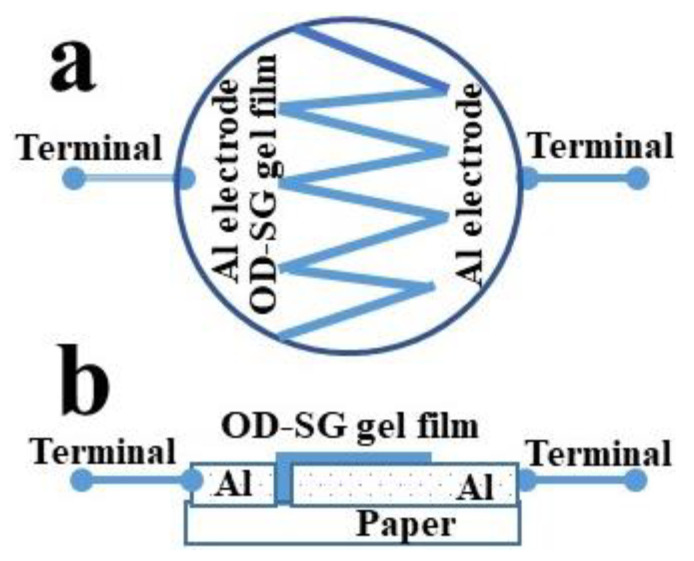
Orange dye and silicone glue gel-based surface type impedimetric and capacitive proximity sensor (**a**) top view and (**b**) front view.

The gel was prepared by mixing OD and silicon glue (1:1 by wt.). As the device performance depends upon the homogeneity of the active layer, the homogeneous mixing of the ingredient of the orange dye (OD) and silicon glue (SG) composite gel was conducted by using a mortar and pestle. Circular-shaped paper substrates with a thickness of 0.1–0.2 mm and diameter of 10 mm were used to fabricate the sensors. Aluminum electrodes of thickness 30 µm were fixed on the paper substrate. The gap b/w the electrodes was in a zig-zag shape. The gap between the two electrodes was 1.0 mm. The prepared orange dye (OD) and silicon glue (SG) composite gel was deposited in the gap using rubbing-in technology. The deposition process in rubbing-in is carried out by fixing the substrate on a solid platform followed by rubbing the gel on the substrate by a well-polished metallic block. The schematic diagram of the rubbing-in process is shown in Figure 8b. The rubbing-in process results in better adhesion of the deposited gel films with the substrates. The thickness of the gel layer was 30 ± 5 µm. The schematic diagram of the fabricated surface-type proximity sensors is shown in Figure 9.

**Figure 10:**
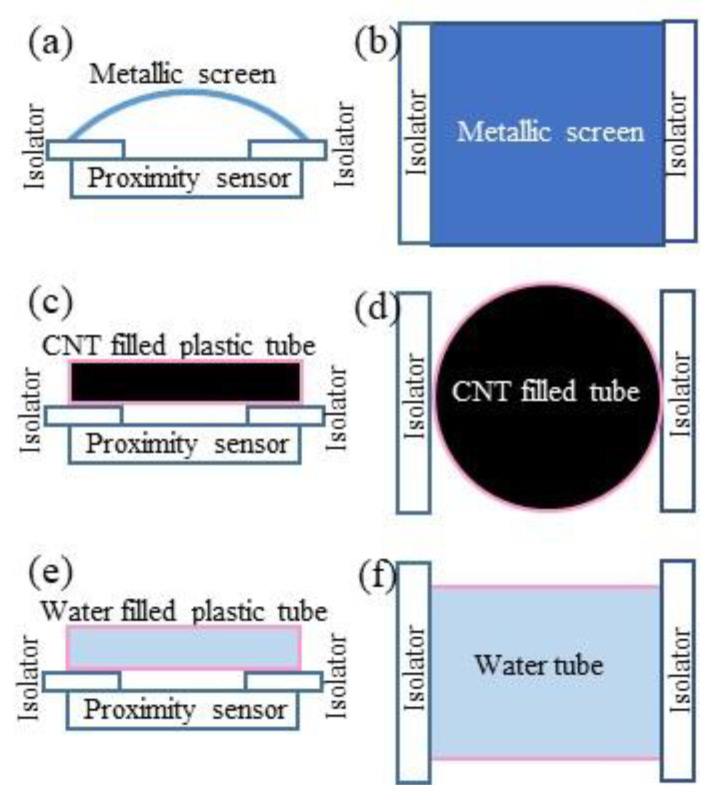
The front and top views of the experimental arrangements for proximity sensing of metallic screen (**a**,**b**), carbon nanotubes (**c**,**d**) and water (**e**,**f**), respectively.

Figure 10 shows the front and top views of the experimental arrangement for the measurement of the proximity effect of the metallic screen (Figure 10a,b), carbon nanotubes (Figure 10c,d) and water (Figure 10e,f), respectively. The semi-cylindrical metallic foil screen (Figure 10a,b), firstly, decreases the negative effect of the electromagnetic noises to the results of the measurements and, secondly, plays the role of the part of variable capacitance. Dielectric plastic variable thickness film-holders (isolators) of the screen allowed the proximity values to be changed by using different thickness plates. Figure 10c,d, respectively, show the front and top views of the experimental setup for the measurement of the CNTs’ proximity. The CNTs’ powder was filled in a “kopeck”-shaped plastic tube, which was used as a receiver. This CNT-filled kopeck-shaped plastic tube was transparent in color. In Figure 10e,f, the cylindrical plastic tube filled with water was used as the receiver. The water filled plastic tube is also transparent.

**Figure 11:**
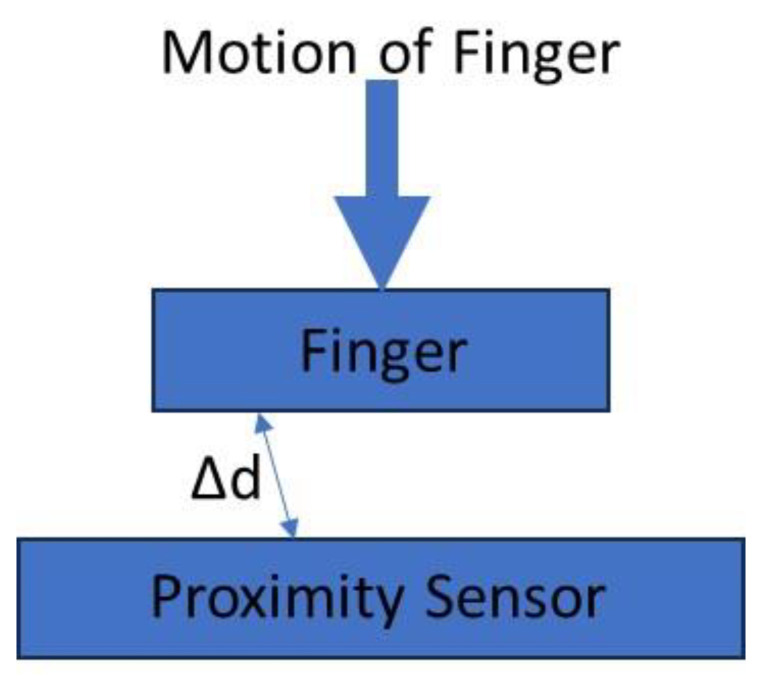
Illustration of the effect of finger on the proximity sensor: Δ*d* is the proximity.

The sensors were also tested under the effect of the finger. Figure 11 shows the schematic diagram illustrating finger proximity sensing.

For the measurement of the impedance and capacitance, a digital meter MT 4090 LCA was used. The utilization of the different heights of the dielectric supports (isolators in Figure 10) allowed the influence of different values of proximity on the impedance and the capacitance of the samples to be investigated.

## Figures and Tables

**Table 1:** The data obtained for various objects in proximity to the fabricated orange dye-silicone glue-based sensors.

Object	Sensing Range (mm)	Frequency (kHz)	Impedimetric Sensing (kΩ/mm)	Capacitive Sensing (10^−2^ pF/mm)
CNT	0.5–3.0	100	59.6	16.8
		200	27.6	19.2
Water	0.5–3.0	100	34.8	18.0
		200	17.2	16.4
Aluminum	0.2–4.0	100	40.2	9.5
		200	24.7	16.3
Finger	0.2–20.0	100	6.2	1.8
		200	2.5	1.5

**Table 2:** The comparison of the fabricated orange dye and silicone glue composite gel-based sensor with the already reported proximity sensors.

Sr.#	Materials	Fabrication Technique	Proximity Range (mm)	Capacitive Sensitivity	Ref.
1	TPU-CNT	Extrusion and pressing	2–12	0–0.48 (dc/c)	[3]
2	Polyacrylamide-CNT-Ag	MBP	0–20	15% (dc/c)	[18]
3	FSR-NM	-----	0 to 7	0.5 pF/mm	[2]
4	TPU-CNT	Extrusion and pressing	3–12	0.5–0.6 (dc/c)	[19]
5	OD-SG-gel	Rubbing-in	0–20	17%	This work

MBP: Mold-bond-polymerize; FSR: Force sensing resistor; NM: Neodymium magnet; OD: Orange dye; SG: Silicone glue; CNT: Carbon nanotubes.

## Data Availability

The data presented in this study are available on request from the corresponding author.
